# Nationwide Multicentric Analysis Regarding In-Hospital Complications After Catheter Ablation of Cardiac Arrhythmias

**DOI:** 10.3390/jcdd13030134

**Published:** 2026-03-11

**Authors:** Florian Doldi, Christian Meyer, Johannes Brachmann, Fabienne Kreimer, Thorsten Lewalter, Roland Tilz, Malte Kuniss, Ibrahim Akin, Philipp Sommer, Thomas Riemer, Jochen Senges, Lars Eckardt

**Affiliations:** 1 Department for Cardiology II: Electrophysiology, University Hospital Münster, Albert-Schweitzer-Campus 1 Gebäude A1, 48149 Münster, Germany; 2 Departement of Cardiology, Angiology, Intensive Care Medicine, EVK Düsseldorf, cNEP, cardiac Neuro- and Electrophysiology Research Consortium, 40217 Düsseldorf, Germany; 3 Department of Cardiology, Angiology and Pulmonology, Klinikum Coburg, 96450 Coburg, Germany; 4 Department of Cardiology and Intensive Unit Care, Hospital Munich South, Peter Osypka Heart Center, 81379 Munich, Germany; 5 Department of Cardiac Electrophysiology, University Hospital Schleswig-Holstein-Campus Lübeck, 23562 Lübeck, Germany; 6 Kerckhoff Heart Center, 61231 Bad Nauheim, Germany; 7 Department of Cardiology, First Department of Medicine, University Medical Center Mannheim, Medical Faculty Mannheim, Heidelberg University, 68167 Mannheim, Germany; 8 Department of Electrophysiology, Heart and Diabetes Center NRW, Ruhr University Bochum, 32545 Bad Oeynhausen, Germany; 9 Stiftung Institut für Herzinfarktforschung (IHF), 67063 Ludwigshafen, Germany

**Keywords:** interventional electrophysiology, catheter ablation, complications, atrial flutter, atrial fibrillation, ventricular tachycardia, multicentric analysis

## Abstract

Objective and Background: With the increasing use of catheter ablation for tachyarrhythmias, continuous evaluation of in-hospital complications is essential. This study aimed at analyzing complications associated with catheter ablation for atrial fibrillation (AF), atrial flutter (AFL), and ventricular tachycardia (VT) using nationwide administrative data. Methods: We conducted a retrospective multicentric data analysis from large German ablation centers between 2018 and 2023. Patients were identified using ICD and OPS codes for AF, AFL, and VT regarding predefined in-hospital complications: mortality, stroke, pericardial tamponade, pulmonary embolism, and vascular complications requiring intervention. Results: Among 19,258 ablation procedures from 11 centers, AF was most common (n = 12,241), followed by AFL (n = 5582) and VT (n = 1435). Major complications occurred in 2.2% (n = 433) of cases. VT ablations had the highest complication rate (9.8%), followed by AF (1.6%) and AFL (1.7%). Pericardial tamponade occurred in 0.9% patients, most commonly in VT ablations (4.0%). Vascular complications requiring intervention were reported in 1.1%, while stroke (0.3%) and pulmonary embolism (0.05%) were rare. In-hospital mortality was highest in VT patients (2.4%), compared to AF (0.08%) and AFL (0.13%). Higher AFL mortality as compared to AF was associated with older age and more comorbidities. Upon exploratory analysis, no statistical association between hospital volume and complication rates could be seen. Conclusions: In this multicenter analysis, catheter ablation was associated with a low overall complication rate. VT ablations carried the highest risk, highlighting the impact of structural heart disease and comorbidities.

## 1. Introduction

Catheter ablation has long been established as a cornerstone in the treatment of cardiac arrhythmias. According to current guidelines, it is considered first-line therapy for paroxysmal supraventricular tachycardias [1], paroxysmal atrial fibrillation [2], and idiopathic ventricular tachycardias [2,3,4]. Growing evidence suggests that early catheter ablation of atrial fibrillation (AF) and ventricular tachycardia (VT) may be associated with prognostic benefit [5,6]. Given these advantages, it is not surprising that the number of catheter ablations performed continuously rises each year [7]. With the increasing number of procedures, the continuous assessment of complications is essential to ensure high-quality healthcare and to facilitate individualized risk stratification for each patient.

The incidences of in-hospital complications vary significantly depending on factors such as the type of arrhythmia, the complexity of the procedure, and the experience of the performing center [8,9]. Reported, partly historical overall complication rates range from 1% to 6% [8,10,11]. Notably, VT ablation seems to be associated with a higher risk profile, as these patients often present with more comorbidities, including heart failure, compared to those undergoing ablation for atrial flutter (AFL) or AF [8,9,10]. To obtain up-to-date insights into complication rates, we aimed at analyzing peri-interventional complications associated with catheter ablation procedures for VT, AF and AFL through an analysis of a nationwide observational registry using administrative data.

## 2. Methods

This study was performed with the participation of the “Institut für Herzinfarktforschung” (IHF) in Ludwigshafen, Germany. We retrospectively analyzed all inpatient cases with documented OPS-code 8-835*, representing catheter ablation procedures, during their hospital stay. Anonymized data from January 2018 until December 2023 were collected from eleven participating centers that provided a complete dataset including complication-related data. These datasets were supplemented annually with the respective §21 data from the preceding year. The datasets were fully anonymized and provided by the medical controlling departments of each center and collected in a nationwide registry. Collected data included baseline characteristics, length and duration of hospital stay, and reason of hospital stay/discharge, as well as the diagnosis (ICD) and procedure (OPS) codes according to the G-DRG System. All data were then compiled into a single dataset, with ICD and OPS codes processed through software packages to classify clinically relevant comorbidities and interventions, as well as defining the type of the treated arrhythmia (Table 1).

The study did not require ethical approval, as it relied solely on anonymized routine data.

## 3. Statistical Analysis

Binary and categorical variables were described by absolute frequencies and percentages, patient age using the mean with standard deviation, and other continuous variables by their medians and quartiles. Differences between groups were assessed with Pearson’s chi-square test or the Wilcoxon test. The relationship between hospital volume and complications during hospitalization was modeled by a generalized linear mixed model. In this model, the logarithms of annual procedure counts were the fixed explanatory variable, and hospitals were considered to have a possible random effect on complication rates, considering repeated measurements over the years. All statistics are based solely on known data, and no imputation was made. Data preparation and statistical analysis for this paper were performed using SAS software (9.4). Copyright © SAS Institute Inc.

## 4. Results

Across 11 participating ablation centers, a total of 19,258 catheter ablations were included. Among these, ablation for therapy of atrial fibrillation (AF) was the most common procedure, accounting for 12,241 cases, followed by atrial flutter (AFL) ablation in 5582 cases and ventricular tachycardia (VT) in 1435 cases. The case–cohort consisted predominantly of elderly male patients (n = 12,786; 66.4%), with 54.1% (n = 10,423) being older than 65 years, with a mean overall age of 64.7 years (±11.5). The median length of hospital stay was 2.0 days (IQR: 1.0, 3.0), with only a small proportion (4.3%; n = 822) requiring intensive care therapy (AF: 2.5%, n = 308; AFL: 3.4%, n = 189; VT: 22.6%, n = 325), with a median stay of 1.8 days (IQR: 0.8, 4.1). Patients receiving AFL ablations were significantly older (66.2 ± 11.5 vs. 64.3 ± 11.0, *p* < 0.01) and had more comorbidities with higher incidences of chronic heart failure (27.3% vs. 17.5%, *p* < 0.01), kidney failure (10.3% vs. 8.3%, *p* < 0.01), diabetes (15.0% vs. 9.3%, *p* < 0.01), hyperlipoproteinemia (28.3% vs. 24.7%, *p* < 0.01), and obesity (9.5% vs. 8.0%, *p* < 0.01) than AF patients (Table 2).

AF ablations were predominantly performed using cryoballoon ablation (n = 7013; 57.3%), while radiofrequency ablation was utilized in 5131 cases (41.9%). In VT ablations, most procedures targeted arrhythmias originating from the left ventricle (n = 1158; 80.7%), with three-dimensional mapping technology being used in 998 cases (69.5%). Epicardial VT ablation was performed in 77 procedures (5.4%). Contact force sensing was used in 23.4% (n = 4498) of all ablations, most frequent in VT procedures (35.2%; n = 505).

In case of atrial flutter ablation, a cavotricuspid isthmus ablation was performed in 80% of cases (n = 4484). Additional or primarily left atrial ablation for atypical flutter was performed in 34.5% (n = 1926) of patients.

Overall, 496 in-hospital complications were recorded after catheter ablation with 433 patients (2.2%) experiencing at least one of the coded complications (excluding multiple coded complications per hospital visit). The highest complication rate (n = 150) was associated with VT ablations (n = 140; 9.8% with at least one of the coded complications) followed by AF ablations (n = 242; patients with at least one of the coded complications: n = 196; 1.6%), and AFL ablations (n = 104; patients with at least one of the coded complications: n = 97; 1.7%).

Pericardial tamponade requiring intervention in association with catheter ablations occurred in 0.9% (n = 168) of cases, with the highest incidence observed in association with VT ablation (n = 57; 4.0% of all VT ablations). Vascular complications necessitating intervention were reported in 1.1% (n = 214) of all patients, with an incidence of 3.1% (n = 45) associated with VT ablations, 1.0% (n = 119) for AF ablations and 0.9% (n = 50) for AFL ablations. Pulmonary arterial embolism was diagnosed in 0.05% (n = 9) of all patients, including 0.2% (n = 3) in association with VT ablations, 0.02% (n = 2) in AF ablations and 0.1% (n = 4) in AFL ablations. Postinterventional stroke was observed in 0.3% (n = 53) of cases, with an incidence of 0.7% (n = 10) for VT ablation, 0.3% (n = 32) for AF ablation and 0.2% (n = 11) for AFL ablation. A total of 52 patients (0.3%) died during the investigated hospital stay. The highest mortality rate was observed in association with a VT ablation procedure, with 35 patients (2.4% of all VT ablations). In contrast, mortality rates were significantly lower for AF (n = 10; 0.08%) and AFL ablations (n = 7; 0.13%) with a *p*-value of <0.01, Table 3.

**Figure 1:**
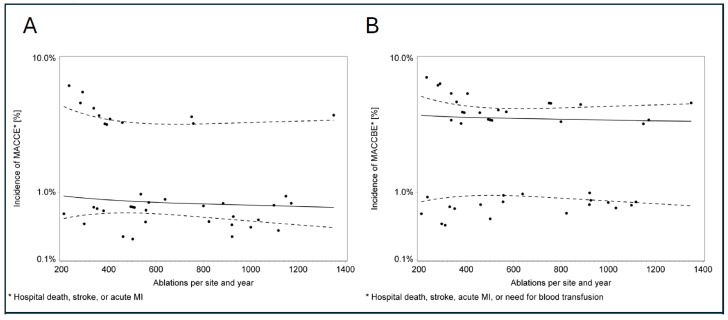
Scatter plot of the association between annual catheter ablation volume and in-hospital complications. Scatter plots illustrating the relationship between yearly catheter ablation volume and complication rates. Each dot represents one centre in a given year; centres may appear multiple times. Analyses were conducted for MACCE (Panel (**A**): in-hospital death, myocardial infarction, stroke) and an extended composite including major bleeding (Panel (**B**): MACCBE). The solid line indicates the linear regression trend, and the dashed lines represent the 95% confidence interval. No association between higher-volume centres and MACCE/MACCBE rates is seen. MACCE = major adverse cardiac and cerebrovascular events; MACCBE = Major Adverse Cardiac, Cerebrovascular and Bleeding Events.

**Figure 2:**
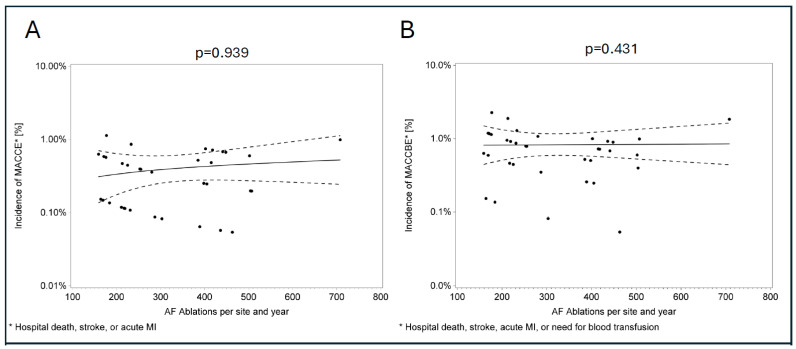
Scatter plot of the association between annual AF ablation volume and in-hospital complications. Scatter plots illustrating the relationship between yearly AF catheter ablation volume per center and complication rates in AF-only ablations. Each dot represents one center in a given year. Panel (**A**): MACCE incidence (in-hospital death, stroke, or acute myocardial infarction (MI)). Panel (**B**): Extended MACCBE incidence (in-hospital death, stroke, acute MI, or need for blood transfusion). Solid line: linear regression trend; dashed lines: 95% confidence interval. No significant association between higher-volume centres and MACCE/MACCBE rates was observed (*p* = 0.939 for MACCE; *p* = 0.431 for MACCBE). MACCE = major adverse cardiac and cerebrovascular events; MACCBE = major adverse cardiac, cerebrovascular, and bleeding.

Analysis of the relationship between hospital volume and in-hospital complications regarding all types of ablation procedures revealed no statistically significant trend (Figure 1). To address potential confounding by heterogeneous ablation types, we performed an exploratory stratified analysis restricted to AF ablations. In a generalized linear mixed model, no significant association between center volume and MACCE (*p* = 0.939) or MACCBE (*p* = 0.431) was observed (Figure 2). Subgroup analyses by energy source also yielded no significant findings (cryoballoon: n = 7013, *p* = 0.32; RF: n = 5131, *p* = 0.20).

Frequency analyses for VT confirmed substantially higher complication risks with epicardial access versus endocardial (MACCE OR = 14.1 [95% CI 6.7–29.9], *p* < 0.001; MACCBE OR = 12.7 [95% CI 6.8–23.8], *p* < 0.001; n = 80 epicardial cases) underscoring procedural complexity as a potential key risk driver beyond volume effects.

## 5. Discussion

Catheter ablation for therapy of tachyarrhythmias is associated with possibly relevant complications [9]. In this study, we present current nationwide, German multicenter administrative data on current in-hospital complications in a large registry of almost 20,000 catheter ablations performed between 2018 and 2023, including cardiac tamponade necessitating drainage, femoral vascular complications requiring intervention, stroke, pulmonary arterial embolism and mortality.

The major findings are the following: (i) complication rates were highest in VT ablation with an overall rate of (≈10%), whereas AF and AFL ablations each remained below 2%; (ii) major complications, i.e., pericardial tamponade or stroke were uncommon with incidences below 1% or 0.5%, respectively; (iii) only few patients died during their hospital stay, with expected highest mortality rate after VT ablations of about 2% but well below 1% for AF and AFL; (iv) the higher AFL mortality compared with AF is most likely due to a much older, more comorbid AFL cohort rather than procedural factors; and (v) no statistical difference in complication rates according to hospital volume could be seen.

Previous studies have consistently shown that the highest complication rates occur in patients undergoing VT ablation, with incidences ranging from 0.6% to 11.2% [8,12,13,14,15], followed by AF ablation (1.2–6.3%) [8,11,16,17,18,19] and AFL ablation (0.1–2.6%) [9,11,20,21,22,23]. Our findings are in line with these older reports, showing overall complication rates of 9.8% (n = 140) for VT ablation, followed by 1.6% (n = 196) for AF ablation and 1.7% (n = 97) for AFL ablation.

One of the most severe peri-interventional complications associated with catheter ablation is pericardial tamponade, with reported incidences ranging from 0.7% to 3.0% [8,9,10,13,24]. Our current data shows a low overall incidence of 0.9%. Vascular complications remain the most frequently observed complications following catheter ablation for cardiac arrhythmias [9,22], with reported incidences depending on the type of procedure and patient characteristics [13,21,22]. The highest incidence of major vascular complications was observed in association with VT ablation, with reported rates ranging from 0.7% to 4.7% [9,15,25], followed by AF ablation (0.5–13.0%) [9,11,18,22] and AFL ablation (<4%) [8,9,11,21,22]. Our findings support earlier observations, with an overall incidence of vascular complications requiring intervention in association with catheter ablations at 1.1%. Further optimization of clinical practices (OAC management) with a progressing widespread use of ultra-sound-guided venous/arterial puncture [26] may further improve the outcome of these patients.

Current post-interventional stroke rates were at the lower end of known incidences in earlier studies. This may be related to an increasing number of ablations performed in the presence of continued oral anticoagulation. Most recent studies have reported stroke incidences ranging from 0% to 0.6% [27,28,29,30,31], with even lower rates observed in AFL ablations (0–0.5%) [8,11,21]. Current guidelines [2,32,33,34], as well as consensus statements [35] recommend performing ablations without interruption of OAC [2,34]. Withholding the morning dose before ablation seems acceptable, as a randomized trial [36] reported comparable safety and efficacy outcomes with minimally interrupted OAC strategy. Our findings are consistent with these reports, showing an overall incidence of post-interventional stroke of 0.3%, with the lowest rate seen in association with AFL ablations (0.2%).

Data on pulmonary embolism (PE) following catheter ablation remain scarce. A recent review and online survey conducted by Burstein et al. [37,38] concluded that PE is fortunately a rare complication after catheter ablation. This finding is further supported by the present analysis. Recent randomized trials also report low PE incidences below 2% following catheter ablation for AF [27] or VT [39]. Furthermore, a recent large observational study [40] analyzing over 45,000 catheter ablations over a 15-year period supports these findings, revealing an overall incidence of just 0.03%, with particularly low rates in AF (0.02%) and AFL (0.0%) ablation procedures. Our analysis aligns closely with these results, showing an overall PE incidence of only 0.05%, with the lowest rate observed in association with AF ablations (0.02%). This may be supported by continuation of oral anticoagulation at the time of the procedure. Given the potentially severe consequences of PE, establishing standardized peri-procedural management strategies, including deep vein thrombosis prophylaxis and optimizing post-interventional patient care, is of paramount importance. However, as data on this subject remains limited and prospective randomized trials are lacking, current guidelines do not yet provide uniform recommendations regarding peri-interventional prophylaxis in these patients [41].

Focusing on in-hospital mortality, previous studies also observed highest rates among patients undergoing VT ablation, with reported incidences ranging from 1.3% to 1.8% [10,12,13,14,20,25]. Our results are coherent to these studies, demonstrating a catheter ablation-associated mortality rate of 2.4%. In comparison, mortality rates were markedly lower in association with AF ablation (0.08%) and AFL ablation (0.13%). The significantly higher overall complication rate, as well as the in-hospital mortality observed in context with VT ablations is most likely attributable to the increased comorbidity burden and the procedural complexity compared to patients requiring only AF or AFL ablation [9,10,20]. Among VT ablations, further descriptive analyses revealed markedly higher complication rates in association with epicardial access versus endocardial (*p* < 0.001), consistent with known procedural hazards [42], despite the limited subgroup size of our cohort (n = 80 epicardial ablation procedures).

Interestingly, as demonstrated in previous studies [9,11], patients undergoing AFL ablation exhibited a higher mortality rate in the same hospital stay than those undergoing AF ablation, despite AFL ablations generally being considered a safe procedure. This phenomenon was also observed in a previous nationwide analysis of administrative data by Steinbeck et al., where mortality rates for AFL ablations were reported to be 3–4 times higher than in patients undergoing AF ablations [11]. The authors hypothesized that these deaths were primarily attributable to pre-existing conditions such as end-stage heart failure, infections, or malignancies rather than the catheter ablation itself, as the procedure was often performed during prolonged hospital stays. A subsequent study [9] was able to confirm this hypothesis through an individual case analysis of administrative data, demonstrating a marked reduction in ablation-associated mortality rates from 0.14% to 0.04% for AFL ablations following individual case assessment. Our study further supports this observation and thereby the limitation of coding-based analyses of complication rates with higher complication rates in patients undergoing AFL versus AF ablations. Patients undergoing AFL ablation were significantly older (AF: 64.3 ± 11.0 years vs. AFL: 66.2 ± 11.5 years, *p* < 0.01) and exhibited more comorbidities, with higher incidences of diabetes, hyperlipoproteinemia, obesity, chronic kidney disease, and chronic heart failure explaining the worse outcome.

In our cohort, the median in-hospital length of stay for AF and AFL ablation was 2 days, reflecting contemporary German inpatient care pathways during the study period rather than dedicated same-day discharge programs. While several centers worldwide have increasingly implemented day-case ablation strategies for selected low-risk patients [16,43,44], our findings illustrate that ablation procedures in Germany are still frequently embedded in more prolonged hospitalizations, which may be driven by comorbidity burden, reimbursement structures, and organizational factors.

Upon exploratory analysis of an association between in-hospital complication rates and procedure volume, our analysis showed no statistical significance among the participating centers (n = 11). Further exploratory subgroup analyses stratified by AF energy source (cryoballoon n = 7013; radiofrequency n = 5131) confirmed the absence of volume–outcome associations (all *p* values > 0.20), suggesting that procedural selection or technical heterogeneity does not confound the primary pooled findings. While these analyses enhance specificity, their exploratory nature, small sample size and reliance on administrative coding limit causal inferences. Given that a recent national survey [7] found only about one quarter of all ablation centers in Germany meet the accreditation criteria as EP training centers defined by the European Heart Rhythm Association (n = 48, 25%; EHRA) and the German Cardiac Society (n = 47, 24%; DGK), this issue warrants further investigation in future studies.

## 6. Limitations

When interpreting our study, several limitations must be considered. Case identification and complication assessment were based on administrative data using ICD and OPS codes, which carries a risk of coding errors and misclassification—particularly for events such as stroke, pericardial tamponade, and in-hospital mortality. Due to the large multicenter dataset, individual case-based validation against clinical records was not feasible; however, German ICD/OPS coding is mandatory and subject to external auditing by health insurance providers, which mitigates but does not eliminate this risk. Reported incidences reflect cumulative OPS and ICD codes per patient, potentially capturing multiple complications during hospitalization. As a retrospective analysis of administrative data lacking individual-level temporal linkage, a direct causal relationship between coded events and catheter ablation cannot be established without case adjudication. Due to the hierarchical multicenter structure, absence of temporal linkage between procedures and events, and potential residual confounding from baseline differences (age, comorbidities, procedural complexity), multivariable regression analyses were deemed inappropriate; comparative findings across AF/AFL/VT groups are therefore presented descriptively only. Additionally, our analysis is restricted to in-hospital events with no follow-up data available, excluding post-discharge complications. Some patients underwent combination procedures (e.g., typical + atypical atrial flutter ablation, or flutter + PVI), meaning complications cannot always be attributed solely to the primary coded arrhythmia. However, as such cases were infrequent, we consider this limitation minor.

## 7. Conclusions

Through the analysis of nearly 20,000 catheter ablations, we report low overall complication rates for catheter ablations for cardiac arrhythmias. VT ablations had the highest complication and mortality rates among all associated procedures. Consistent with prior administrative data analyses, in-hospital mortality in association with AFL ablation was paradoxically higher than after AF ablation, likely influenced by patient comorbidities rather than the procedure itself. Although thromboembolic complications, including stroke and pulmonary embolism, remain rare, further studies are needed to optimize peri-interventional management, particularly regarding oral anticoagulation and prophylaxis of venous thrombosis. Additionally, we performed an exploratory analysis of the volume–outcome relationship between the 11 participating centers and could not find a statistically significant relationship between complication rates and hospital procedural volume. Continuous monitoring, improved risk stratification, and standardized peri-procedural management—along with efforts to define and meet volume and accreditation benchmarks—may be essential to further improve the safety and efficacy of catheter ablation procedures.

## Figures and Tables

**Table 1:** Classification of ICD/OPS codes defining the type of treated arrhythmia and complication.

Condition/Complication	ICD-10-GM/OPS Codes
Atrial fibrillation (AF)	I48.0; I48.1; I48.2
Atrial flutter (typical and atypical; AFL)	I48.3; I48.4
Ventricular tachycardia (VT)	I49.3; I49.4
Pericardial tamponade	5-370.0; 8-152.0; 5-374.08
Vascular complication requiring intervention	5-38.x; 5-39.x; 8-836
Stroke	I63.x; I64; I69.4; 8-98x
Pulmonary arterial embolism	I26.0; I26.9
Death (in-hospital)	R95–R99; I46.1

**Table 2:** Baseline and procedural characteristics of all included patients either receiving a catheter ablation for AF, AFL or VT.

		Overall	AF	AFL	VT	*p*-Value
Baseline Characteristics						
	Total number of ablations	19,258	12,241	5582	1435	
	Number of coded complications (%) *	496	242	104	150	<0.01
	Cases with at least one of the coded complications (%)	433 (2.2)	196 (1.6)	97 (1.7)	140 (9.8)	<0.01
	Age (y; mean ± SD)	64.7 (±11.5)	64.3 (±11.0)	66.2 (±11.5)	62.1 (±14.1)	<0.01
	Age > 65 years (%)	10,423 (54.1)	6413 (52.4)	3330 (59.7)	680 (47.4)	<0.01
	Sex (male (%))	12,786 (66.4)	7640 (62.4)	3995 (71.6)	1151 (80.2)	<0.01
	Length of Stay (days; Quartiles)	2.0 (1.0; 3.0)	2.0 (1.0; 3.0)	2.0 (1.0; 4.0)	5.0 (1.0; 9.0)	<0.01
Comorbidities						
	Ischemic Cardiomyopathy (%)	4571 (23.7)	2276 (18.6)	1416 (25.4)	879 (61.3)	<0.01
	Chronic Heart Failure (%)	4544 (23.6)	2139 (17.5)	1522 (27.3)	883 (61.5)	<0.01
	Arterial Hypertension (%)	12,434 (64.6)	7953 (65.0)	3602 (64.5)	879 (61.3)	0.02
	Diabetes Mellitus (%)	2219 (11.5)	1133 (9.3)	837 (15.0)	249 (17.4)	<0.01
	Stroke (%)	49 (0.3)	30 (0.3)	10 (0.2)	9 (0.6)	<0.01
	Obesity (%)	1591 (8.3)	975 (8.0)	532 (9.5)	84 (5.9)	<0.01
	Hyperlipoproteinemia (%)	5200 (27.0)	3026 (24.7)	1577 (28.3)	597 (41.6)	<0.01
	Chronic obstructive pulmonary disease (%)	623 (3.2)	318 (2.6)	231 (4.1)	74 (5.2)	<0.01
	Chronic kidney insufficiency (%)	1882 (9.8)	1017 (8.3)	575 (10.3)	290 (20.2)	<0.01
Mapping/Ablation						
Location of Ablation	Right Atrium (%)	5656 (29.4)	1133 (9.3)	4484 (80.0)	39 (2.7)	<0.01
	Left Atrium/PV (%)	14,172 (73.6)	12,241 (100.0)	1926 (34.5)	5 (0.4)	<0.01
	Right Ventricle (%)	624 (3.2)	27 (0.2)	193 (3.5)	404 (28.2)	<0.01
	Left Ventricle (%)	1223 (6.4)	50 (0.4)	15 (0.3)	1158 (80.7)	<0.01
	Epicardial (%)	80 (0.4)	3 (0.02)	0 (0.0)	77 (5.4)	<0.01
Ablation method	Radiofrequency (%)	11,877 (61.7)	5131 (41.9)	5311 (95.2)	1435 (100.0)	<0.01
	Cryoballoon (%)	7626 (39.0)	7013 (57.3)	612 (11.0)	1 (0.1)	<0.01
	3D-Mapping technology (%)	6027 (31.3)	3223 (26.3)	1806 (32.4)	998 (69.5)	<0.01
	Contact Force Measurement (%)	4498 (23.4)	2839 (23.2)	1154 (20.7)	505 (35.2)	<0.01

Cumulative percentages do not always add up to 100%, as some patients received simultaneous ablations of arrhythmias of different type or origin. * This reflects the true cumulative sum of all coded in-hospital complications (including multiple complications per patient).

**Table 3:** Patients suffering from an in-hospital complication after catheter ablation of either AF, AFL, or VT.

	Overall	AF	AFL	VT	*p*-Value
Total number of coded complications (%) *	496	242	104	150	
Cases with at least one of the coded complications (%)	433 (2.2)	196 (1.6)	97 (1.7)	140 (9.8)	<0.01
Pericardial Tamponade (%)	168 (0.9)	79 (0.7)	32 (0.6)	57 (4.0)	<0.01
Vascular Complication (%)	214 (1.1)	119 (1.0)	50 (0.9)	45 (3.1)	<0.01
Pulmonary arterial embolism (%)	9 (0.05)	2 (0.02)	4 (0.1)	3 (0.2)	<0.01
Stroke (%)	53 (0.3)	32 (0.3)	11 (0.2)	10 (0.7)	<0.01
Death (%)	52 (0.3)	10 (0.08)	7 (0.13)	35 (2.4)	<0.01

Cumulative percentages of complication rates do not always add up to 100%, as some patients experienced several complications in the setting of their hospital stay. * This reflects the true cumulative sum of all coded in-hospital complications (including multiple complications per patient).

## Data Availability

Data available on request.

## References

[1] Brugada J., Katritsis D.G., Arbelo E., Arribas F., Bax J.J., Blomstrom-Lundqvist C., Calkins H., Corrado D., Deftereos S.G., Diller G.P. (2020). 2019 ESC Guidelines for the management of patients with supraventricular tachycardiaThe Task Force for the management of patients with supraventricular tachycardia of the European Society of Cardiology (ESC). Eur. Heart J..

[2] Van Gelder I.C., Rienstra M., Bunting K.V., Casado-Arroyo R., Caso V., Crijns H.J.G.M., De Potter T.J.R., Dwight J., Guasti L., Hanke T. (2024). 2024 ESC Guidelines for the management of atrial fibrillation developed in collaboration with the European Association for Cardio-Thoracic Surgery (EACTS): Developed by the task force for the management of atrial fibrillation of the European Society of Cardiology (ESC), with the special contribution of the European Heart Rhythm Association (EHRA) of the ESC. Endorsed by the European Stroke Organisation (ESO). Eur. Heart J..

[3] Könemann H., Dagres N., Merino J.L., Sticherling C., Zeppenfeld K., Tfelt-Hansen J., Eckardt L. (2023). Spotlight on the 2022 ESC guideline management of ventricular arrhythmias and prevention of sudden cardiac death: 10 novel key aspects. Europace.

[4] Zeppenfeld K., Tfelt-Hansen J., de Riva M., Winkel B.G., Behr E.R., Blom N.A., Charron P., Corrado D., Dagres N., de Chillou C. (2022). 2022 ESC Guidelines for the management of patients with ventricular arrhythmias and the prevention of sudden cardiac death. Eur. Heart J..

[5] Romero J., Di Biase L., Diaz J.C., Quispe R., Du X., Briceno D., Avendano R., Tedrow U., John R.M., Michaud G.F. (2018). Early Versus Late Referral for Catheter Ablation of Ventricular Tachycardia in Patients with Structural Heart Disease: A Systematic Review and Meta-Analysis of Clinical Outcomes. JACC Clin. Electrophysiol..

[6] Frankel D.S., Mountantonakis S.E., Robinson M.R., Zado E.S., Callans D.J., Marchlinski F.E. (2011). Ventricular tachycardia ablation remains treatment of last resort in structural heart disease: Argument for earlier intervention. J. Cardiovasc. Electrophysiol..

[7] Eckardt L., Doldi F., Busch S., Duncker D., Estner H., Kuniss M., Metzner A., Meyer C., Neuberger H.R., Tilz R. (2022). 10-year follow-up of interventional electrophysiology: Updated German survey during the COVID-19 pandemic. Clin. Res. Cardiol..

[8] Hosseini S.M., Rozen G., Saleh A., Vaid J., Biton Y., Moazzami K., Heist E.K., Mansour M.C., Kaadan M.I., Vangel M. (2017). Catheter Ablation for Cardiac Arrhythmias: Utilization and In-Hospital Complications, 2000 to 2013. JACC Clin. Electrophysiol..

[9] Eckardt L., Doldi F., Anwar O., Gessler N., Scherschel K., Kahle A.K., von Falkenhausen A.S., Thaler R., Wolfes J., Metzner A. (2023). Major in-hospital complications after catheter ablation of cardiac arrhythmias: Individual case analysis of 43 031 procedures. Europace.

[10] Peichl P., Wichterle D., Pavlu L., Cihak R., Aldhoon B., Kautzner J. (2014). Complications of catheter ablation of ventricular tachycardia: A single-center experience. Circ. Arrhythm. Electrophysiol..

[11] Steinbeck G., Sinner M.F., Lutz M., Müller-Nurasyid M., Kääb S., Reinecke H. (2018). Incidence of complications related to catheter ablation of atrial fibrillation and atrial flutter: A nationwide in-hospital analysis of administrative data for Germany in 2014. Eur. Heart J..

[12] Palaniswamy C., Kolte D., Harikrishnan P., Khera S., Aronow W.S., Mujib M., Mellana W.M., Eugenio P., Lessner S., Ferrick A. (2014). Catheter ablation of postinfarction ventricular tachycardia: Ten-year trends in utilization, in-hospital complications, and in-hospital mortality in the United States. Heart Rhythm.

[13] Tilz R.R., Lin T., Eckardt L., Deneke T., Andresen D., Wieneke H., Brachmann J., Kääb S., Chun K.R.J., Münkler P. (2018). Ablation Outcomes and Predictors of Mortality Following Catheter Ablation for Ventricular Tachycardia: Data from the German Multicenter Ablation Registry. J. Am. Heart Assoc..

[14] Mathew S., Fink T., Feickert S., Inaba O., Hashiguchi N., Schlüter M., Wohlmuth P., Wissner E., Tilz R.R., Heeger C.H. (2022). Complications and mortality after catheter ablation of ventricular arrhythmias: Risk in VT ablation (RIVA) score. Clin. Res. Cardiol..

[15] König S., Ueberham L., Müller-Rothing R., Wiedemann M., Ulbrich M., Sause A., Tebbenjohanns J., Schade A., Shin D.I., Staudt A. (2020). Catheter ablation of ventricular arrhythmias and in-hospital mortality: Insights from the German-wide Helios hospital network of 5052 cases. Europace.

[16] König S., Andrade J.G., Bollmann A. (2022). Administrative data confirm safety of same-day discharge following catheter ablation of atrial fibrillation: All good or is there a fly in the ointment?. EP Eur..

[17] König S., Ueberham L., Schuler E., Wiedemann M., Reithmann C., Seyfarth M., Sause A., Tebbenjohanns J., Schade A., Shin D.I. (2018). In-hospital mortality of patients with atrial arrhythmias: Insights from the German-wide Helios hospital network of 161 502 patients and 34 025 arrhythmia-related procedures. Eur. Heart J..

[18] Deshmukh A., Patel N.J., Pant S., Shah N., Chothani A., Mehta K., Grover P., Singh V., Vallurupalli S., Savani G.T. (2013). In-Hospital Complications Associated with Catheter Ablation of Atrial Fibrillation in the United States Between 2000 and 2010. Circulation.

[19] Benali K., Khairy P., Hammache N., Petzl A., Da Costa A., Verma A., Andrade J.G., Macle L. (2023). Procedure-Related Complications of Catheter Ablation for Atrial Fibrillation. J. Am. Coll. Cardiol..

[20] Pastapur A., McBride D., Deshmukh A., Driesenga S., Ghannam M., Bogun F., Liang J.J. (2023). Complications of catheter ablation for ventricular tachycardia. J. Interv. Card. Electrophysiol..

[21] Dechering D.G., Gonska B.D., Brachmann J., Lewalter T., Kuck K.H., Andresen D., Willems S., Spitzer S.G., Straube F., Schumacher B. (2021). Efficacy and complications of cavo-tricuspid isthmus-dependent atrial flutter ablation in patients with and without structural heart disease: Results from the German Ablation Registry. J. Interv. Card. Electrophysiol..

[22] Bohnen M., Stevenson W.G., Tedrow U.B., Michaud G.F., John R.M., Epstein L.M., Albert C.M., Koplan B.A. (2011). Incidence and predictors of major complications from contemporary catheter ablation to treat cardiac arrhythmias. Heart Rhythm.

[23] Perez F.J., Schubert C.M., Parvez B., Pathak V., Ellenbogen K.A., Wood M.A. (2009). Long-term outcomes after catheter ablation of cavo-tricuspid isthmus dependent atrial flutter: A meta-analysis. Circ. Arrhythm. Electrophysiol..

[24] Doldi F., Doldi P.M., Plagwitz L., Westerwinter M., Wolfes J., Korthals D., Willy K., Wegner F.K., Könemann H., Ellermann C. (2023). Predictors for major in-hospital complications after catheter ablation of ventricular arrhythmias: Validation and modification of the Risk in Ventricular Ablation (RIVA) Score. Clin. Res. Cardiol..

[25] Ding W.Y., Pearman C.M., Bonnett L., Adlan A., Chin S.H., Denham N., Modi S., Todd D., Hall M.C.S., Mahida S. (2022). Complication rates following ventricular tachycardia ablation in ischaemic and non-ischaemic cardiomyopathies: A systematic review. J. Interv. Card. Electrophysiol..

[26] Sharma P.S., Padala S.K., Gunda S., Koneru J.N., Ellenbogen K.A. (2016). Vascular Complications During Catheter Ablation of Cardiac Arrhythmias: A Comparison Between Vascular Ultrasound Guided Access and Conventional Vascular Access. J. Cardiovasc. Electrophysiol..

[27] Packer D.L., Mark D.B., Robb R.A., Monahan K.H., Bahnson T.D., Poole J.E., Noseworthy P.A., Rosenberg Y.D., Jeffries N., Mitchell L.B. (2019). Effect of Catheter Ablation vs Antiarrhythmic Drug Therapy on Mortality, Stroke, Bleeding, and Cardiac Arrest Among Patients with Atrial Fibrillation: The CABANA Randomized Clinical Trial. JAMA.

[28] Andrade J.G., Wells G.A., Deyell M.W., Bennett M., Essebag V., Champagne J., Roux J.F., Yung D., Skanes A., Khaykin Y. (2021). Cryoablation or Drug Therapy for Initial Treatment of Atrial Fibrillation. N. Engl. J. Med..

[29] Wazni O.M., Dandamudi G., Sood N., Hoyt R., Tyler J., Durrani S., Niebauer M., Makati K., Halperin B., Gauri A. (2021). Cryoballoon Ablation as Initial Therapy for Atrial Fibrillation. N. Engl. J. Med..

[30] Reddy V.Y., Gerstenfeld E.P., Natale A., Whang W., Cuoco F.A., Patel C., Mountantonakis S.E., Gibson D.N., Harding J.D., Ellis C.R. (2023). Pulsed Field or Conventional Thermal Ablation for Paroxysmal Atrial Fibrillation. N. Engl. J. Med..

[31] Schmidt B., Bordignon S., Neven K., Reichlin T., Blaauw Y., Hansen J., Adelino R., Ouss A., Futing A., Roten L. (2023). EUropean real-world outcomes with Pulsed field ablatiOn in patients with symptomatic atRIAl fibrillation: Lessons from the multi-centre EU-PORIA registry. Europace.

[32] Steffel J., Collins R., Antz M., Cornu P., Desteghe L., Haeusler K.G., Oldgren J., Reinecke H., Roldan-Schilling V., Rowell N. (2021). 2021 European Heart Rhythm Association Practical Guide on the Use of Non-Vitamin K Antagonist Oral Anticoagulants in Patients with Atrial Fibrillation. Europace.

[33] Nogami A., Kurita T., Kusano K., Goya M., Shoda M., Tada H., Naito S., Yamane T., Kimura M., Shiga T. (2022). JCS/JHRS 2021 Guideline Focused Update on Non-Pharmacotherapy of Cardiac Arrhythmias. Circ. J..

[34] Joglar J.A., Chung M.K., Armbruster A.L., Benjamin E.J., Chyou J.Y., Cronin E.M., Deswal A., Eckhardt L.L., Goldberger Z.D., Gopinathannair R. (2024). 2023 ACC/AHA/ACCP/HRS Guideline for the Diagnosis and Management of Atrial Fibrillation: A Report of the American College of Cardiology/American Heart Association Joint Committee on Clinical Practice Guidelines. Circulation.

[35] Calkins H., Hindricks G., Cappato R., Kim Y.H., Saad E.B., Aguinaga L., Akar J.G., Badhwar V., Brugada J., Camm J. (2018). 2017 HRS/EHRA/ECAS/APHRS/SOLAECE expert consensus statement on catheter and surgical ablation of atrial fibrillation. Europace.

[36] van Vugt S.P.G., Westra S.W., Volleberg R., Hannink G., Nakamura R., de Asmundis C., Chierchia G.B., Navarese E.P., Brouwer M.A. (2021). Meta-analysis of controlled studies on minimally interrupted vs. continuous use of non-vitamin K antagonist oral anticoagulants in catheter ablation for atrial fibrillation. Europace.

[37] Burstein B., Barbosa R.S., Kalfon E., Joza J., Bernier M., Essebag V. (2017). Venous Thrombosis After Electrophysiology Procedures: A Systematic Review. Chest.

[38] Burstein B., Barbosa R.S., Samuel M., Kalfon E., Philippon F., Birnie D., Mangat I., Redfearn D., Sandhu R., Macle L. (2018). Prevention of venous thrombosis after electrophysiology procedures: A survey of national practice. J. Interv. Card. Electrophysiol..

[39] Reddy V.Y., Reynolds M.R., Neuzil P., Richardson A.W., Taborsky M., Jongnarangsin K., Kralovec S., Sediva L., Ruskin J.N., Josephson M.E. (2007). Prophylactic catheter ablation for the prevention of defibrillator therapy. N. Engl. J. Med..

[40] Doldi F., Geßler N., Anwar O., Kahle A.-K., Scherschel K., Rath B., Köbe J., Lange P.S., Frommeyer G., Metzner A. (2024). In-Hospital Pulmonary Arterial Embolism after Catheter Ablation of Over 45,000 Cardiac Arrhythmias: Individualized Case Analysis of Multicentric Data. Thromb. Haemost..

[41] Mugnai G., Farkowski M., Tomasi L., Roten L., Migliore F., de Asmundis C., Conte G., Boveda S., Chun J.K.R. (2023). Prevention of venous thromboembolism in right heart-sided electrophysiological procedures: Results of an European Heart Rhythm Association survey. Europace.

[42] Müller J., Chakarov I., Halbfass P., Nentwich K., Berkovitz A., Sonne K., Barth S., Lehrmann H., Deneke T. (2025). Epicardial ventricular tachycardia ablation: Safety and efficacy of access and ablation using low-iodine content. Clin. Res. Cardiol..

[43] Ignacio D.M., Jarma D.J.J., Nicolas V., Gustavo D., Leandro T., Milagros C., Vasquez E., Alberto G., Santiago R., Gaston A. (2018). Current Safety of Pulmonary Vein Isolation in Paroxysmal Atrial Fibrillation: First Experience of Same Day Discharge. J. Atr. Fibrillation.

[44] Rajendra A., Osorio J., Diaz J.C., Hoyos C., Rivera E., Matos C.D., Costea A., Varley A.L., Thorne C., Hoskins M. (2023). Performance of the REAL-AF Same-Day Discharge Protocol in Patients Undergoing Catheter Ablation of Atrial Fibrillation. JACC Clin. Electrophysiol..

